# 2,5-Bis{[(–)-(*S*)-1-(4-methyl­phen­yl)eth­yl]imino­meth­yl}thio­phene

**DOI:** 10.1107/S1600536813021685

**Published:** 2013-08-14

**Authors:** Sylvain Bernès, Guadalupe Hernández-Téllez, Manju Sharma, Oscar Portillo-Moreno, René Gutiérrez

**Affiliations:** a DEP Facultad de Ciencias Químicas, UANL, Guerrero y Progreso S/N, Col. Treviño, 64570 Monterrey, NL, Mexico; b Laboratorio de Síntesis de Complejos, Facultad de Ciencias Químicas, Universidad Autónoma de Puebla, PO Box 156, 72001 Puebla, Pue., Mexico; c Ingeniería Bioquímica, Instituto Tecnológico Superior de Atlixco, 74218 Atlixco, Pue., Mexico

## Abstract

The title chiral bis-aldimine, C_24_H_26_N_2_S, was synthesized using a solvent-free Schiff condensation. The mol­ecule displays crystallographic *C*_2_ symmetry, with the S atom lying on the twofold axis parallel to [100]. As a consequence of the (*S*,*S*) stereochemistry, the tolyl groups are oriented towards opposite faces of the thiophene core, giving a twisted conformation for the whole mol­ecule. Mol­ecules are arranged in the crystal in a herringbone-like pattern, without any significant inter­molecular contacts.

## Related literature

For the solvent-free approach in organic synthesis, see: Tanaka & Toda (2000 ▶). For the structure of a chiral bis-aldimine compound, see: Espinosa Leija *et al.* (2009 ▶). For structures of thio­phenes substituted in positions 2 and 5 by imine functionalities, see: Skene & Dufresne (2006 ▶); Fridman & Kaftory (2007 ▶); de Lima *et al.* (2010 ▶); Kudyakova *et al.* (2011 ▶, 2012 ▶).
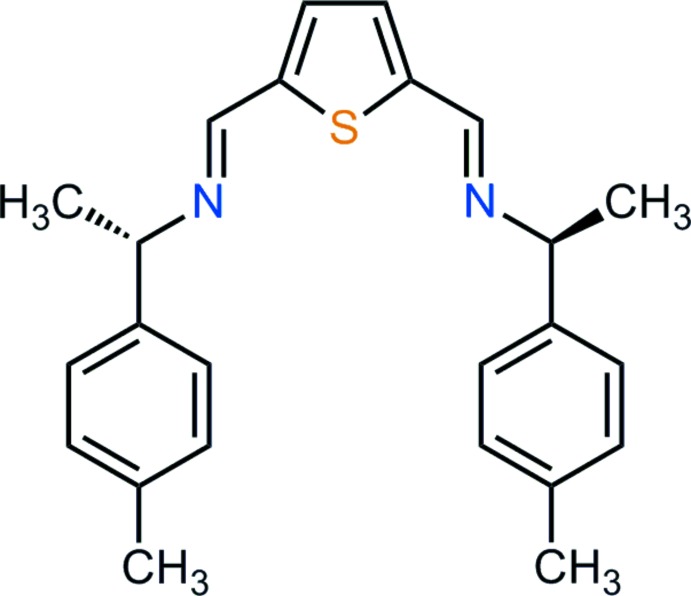


## Experimental

### 

#### Crystal data

C_24_H_26_N_2_S
*M* *_r_* = 374.53
Orthorhombic, 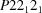

*a* = 6.278 (3) Å
*b* = 7.900 (3) Å
*c* = 21.500 (7) Å
*V* = 1066.4 (7) Å^3^
*Z* = 2
Mo *K*α radiation
μ = 0.16 mm^−1^
*T* = 298 K
0.50 × 0.32 × 0.10 mm

#### Data collection

Bruker P4 diffractometer
Absorption correction: ψ scan (*XSCANS*; Siemens, 1996 ▶) *T*_min_ = 0.754, *T*_max_ = 0.985
3046 measured reflections
1767 independent reflections
1352 reflections with *I* > 2σ(*I*)
*R*_int_ = 0.066
3 standard reflections every 97 reflections intensity decay: 2.5%

#### Refinement

*R*[*F* ^2^ > 2σ(*F* ^2^)] = 0.052
*wR*(*F* ^2^) = 0.155
*S* = 1.06
1767 reflections
125 parameters
H-atom parameters constrained
Δρ_max_ = 0.22 e Å^−3^
Δρ_min_ = −0.26 e Å^−3^
Absolute structure: Flack x determined using 412 quotients [(I+)-(I-)]/[(I+)+(I-)] (Parsons & Flack, 2004 ▶)
Absolute structure parameter: −0.08 (18)

### 

Data collection: *XSCANS* (Siemens, 1996 ▶); cell refinement: *XSCANS*; data reduction: *XSCANS*; program(s) used to solve structure: *SHELXS2013* (Sheldrick, 2008 ▶); program(s) used to refine structure: *SHELXL2013* (Sheldrick, 2008 ▶); molecular graphics: *Mercury* (Macrae *et al.*, 2008 ▶); software used to prepare material for publication: *SHELXL2013*.

## Supplementary Material

Crystal structure: contains datablock(s) I, global. DOI: 10.1107/S1600536813021685/nc2315sup1.cif

Structure factors: contains datablock(s) I. DOI: 10.1107/S1600536813021685/nc2315Isup2.hkl

Supplementary material file. DOI: 10.1107/S1600536813021685/nc2315Isup3.cml

Additional supplementary materials:  crystallographic information; 3D view; checkCIF report

## Figures and Tables

**Fig. 1.**
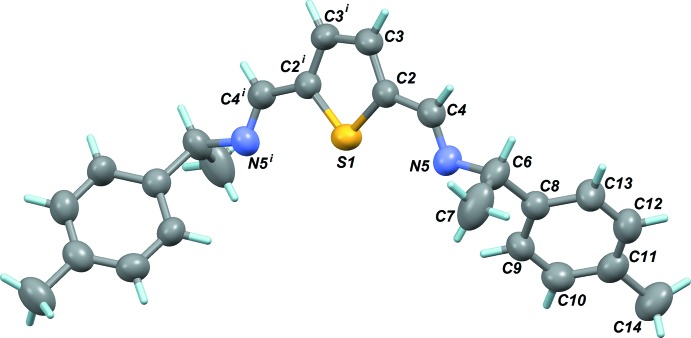
The title molecule with displacement ellipsoids for non-H atoms shown at the 50% probability level. The asymmetric unit consists of half of the molecule, and symmetry code to generate equivalent atoms is i = x, 1 - y, 2 - z.

**Fig. 2.**
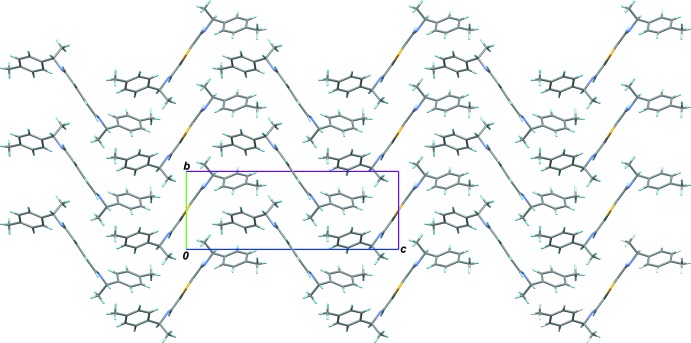
Part of the crystal structure, viewed along the a-axis.

## supplementary crystallographic information

### 1. Comment

In the last few years, our attention has been focused on the synthesis and structure of chiral bis-imines (*e.g*. Espinosa Leija *et al.*, 2009), mostly due to their versatile coordination behavior and interesting properties as ligands for building of chiral metal complexes. Along this line, the title compound was synthesized through a Schiff condensation between a low-melting point dialdehyde and a liquid amine having a high boiling-point (above 200 °C), which also serves as a solvent for the reaction. No other solvents were used for the reaction (Tanaka & Toda, 2000).

The crude compound crystallized from CH_2_Cl_2_, allowing to determine its chiral purity and crystal structure. The obtained bis-aldimine is the expected (*S*,*S*) diastereoisomer, with imine bonds in the common *E* configuration (Fig. 1). The molecule is placed on the 2-fold axis of space group *P*22_1_2_1_, with the S atom lying on the symmetry axis. The resulting molecular conformation displays the *C*_2_ symmetry, with imine arms oriented towards opposite sides of the central thiophene core ring. Other thiophenes substituted in positions 2 and 5 by imine groups have been characterized (*e.g*. Skene & Dufresne, 2006; Fridman & Kaftory, 2007; de Lima *et al.*, 2010; Kudyakova *et al.*, 2011, 2012). However, all were achiral compounds, and only one actually presented a crystallographic *C*_2_ symmetry (space group *C*2/*c*, Kudyakova *et al.*, 2011), as in the title compound.

The molecules are arranged in the crystal in such a way they form a herringbone-like structure (Fig. 2). However, no actual supramolecular pattern is formed in the solid-state, since no intermolecular contacts of significant strength are present.

### 2. Experimental

Under solvent-free conditions, a mixture of 2,5-thiophenedicarboxaldehyde (100 mg, 0.71 mmol) and (*S*)-(–)-1-(4-methylphenyl)ethylamine (192 mg, 1.42 mmol) in a 1:2 molar ratio were mixed at room temperature, giving a brownish solid. The crude was recrystallized from CH_2_Cl_2_, affording colorless crystals of the title compound, in 91% yield. *M*.p. 150–152 °C. Spectroscopic data: [α]^25^_D_ = +59.9 (c 1, CHCl_3_). IR (KBr): 1624 cm^-1^ (C═N). ^1^H-NMR (400 MHz, CDCl_3_/TMS, p.p.m.): δ = 1.54–1.55 (d, 6H, CHC*H*_3_), 2.33 (s, 6H, PhC*H*_3_), 4.46, 4.51 (q, 2H, C*H*), 7.13–7.29 (m, 10H, Ar), 8.34 (s, 2H, *H*C═N). ^13^C-NMR (100 MHz, CDCl_3_/TMS, p.p.m.): δ = 21.0 (C*C*H_3_), 30.9 (Ph*C*H_3_), 69.0 (*C*HCH_3_), 126.5 (Ar), 129.0 (Ar), 129.7 (Ar), 136.4 (Ar), 141.9 (Ar), 145.1 (Ar), 152.2 (H*C*═N). MS—EI: *m*/*z* = 374 (*M*^+^).

### 3. Refinement

All C-bonded H atoms were placed in idealized positions and refined as riding to their carrier C atoms, with bond lengths fixed to 0.93 (aromatic CH), 0.96 (methyl CH_3_), and 0.98 Å (methine CH). Isotropic displacement parameters were calculated as *U*_iso_(H) = 1.5*U*_eq_(C7, C14) for methyl groups and *U*_iso_(H) = 1.2*U*_eq_(carrier C) for other H atoms. The absolute configuration was assigned from the known configuration of the chiral amine used as starting material. The absolute structure was confirmed through the Parsons-Flack test (Parsons & Flack, 2004).

### Crystal data

**Table:**

C_24_H_26_N_2_S	*D*_x_ = 1.166 Mg m^−^^3^
*M**_r_* = 374.53	Melting point: 423 K
Orthorhombic, *P*22_1_2_1_	Mo *K*α radiation, λ = 0.71073 Å
Hall symbol: P 2bc 2	Cell parameters from 71 reflections
*a* = 6.278 (3) Å	θ = 4.2–12.3°
*b* = 7.900 (3) Å	µ = 0.16 mm^−^^1^
*c* = 21.500 (7) Å	*T* = 298 K
*V* = 1066.4 (7) Å^3^	Plate, colourless
*Z* = 2	0.50 × 0.32 × 0.10 mm
*F*(000) = 400	

### Data collection

**Table:**

Bruker P4 diffractometer	1352 reflections with *I* > 2σ(*I*)
Radiation source: fine-focus sealed tube	*R*_int_ = 0.066
Graphite monochromator	θ_max_ = 25.1°, θ_min_ = 1.9°
2θ/ω scans	*h* = −7→5
Absorption correction: ψ scan (*XSCANS*; Siemens, 1996)	*k* = −9→9
*T*_min_ = 0.754, *T*_max_ = 0.985	*l* = −25→25
3046 measured reflections	3 standard reflections every 97 reflections
1767 independent reflections	intensity decay: 2.5%

### Refinement

**Table:**

Refinement on *F*^2^	Secondary atom site location: difference Fourier map
Least-squares matrix: full	Hydrogen site location: inferred from neighbouring sites
*R*[*F*^2^ > 2σ(*F*^2^)] = 0.052	H-atom parameters constrained
*wR*(*F*^2^) = 0.155	*w* = 1/[σ^2^(*F*_o_^2^) + (0.0559*P*)^2^ + 0.760*P*] where *P* = (*F*_o_^2^ + 2*F*_c_^2^)/3
*S* = 1.06	(Δ/σ)_max_ < 0.001
1767 reflections	Δρ_max_ = 0.22 e Å^−^^3^
125 parameters	Δρ_min_ = −0.26 e Å^−^^3^
0 restraints	Absolute structure: Flack x determined using 412 quotients [(I+)-(I-)]/[(I+)+(I-)] (Parsons & Flack, 2004)
0 constraints	Absolute structure parameter: −0.08 (18)
Primary atom site location: structure-invariant direct methods	

### Fractional atomic coordinates and isotropic or equivalent isotropic displacement parameters (Å^2^)

**Table:**

	*x*	*y*	*z*	*U*_iso_*/*U*_eq_	
S1	0.6440 (3)	0.5000	1.0000	0.0580 (5)	
C2	0.8347 (8)	0.3700 (6)	0.9686 (2)	0.0536 (12)	
C3	1.0327 (8)	0.4251 (7)	0.9825 (2)	0.0645 (15)	
H3B	1.1562	0.3696	0.9701	0.077*	
C4	0.7765 (9)	0.2268 (7)	0.9303 (2)	0.0579 (13)	
H4A	0.8834	0.1535	0.9172	0.069*	
N5	0.5897 (7)	0.1971 (6)	0.91410 (19)	0.0578 (11)	
C6	0.5569 (9)	0.0468 (7)	0.8745 (2)	0.0651 (15)	
H6B	0.6951	0.0064	0.8594	0.078*	
C7	0.4544 (14)	−0.0898 (8)	0.9141 (3)	0.099 (2)	
H7B	0.5485	−0.1196	0.9476	0.148*	
H7C	0.4271	−0.1879	0.8890	0.148*	
H7D	0.3226	−0.0483	0.9309	0.148*	
C8	0.4176 (8)	0.0904 (6)	0.8199 (2)	0.0540 (12)	
C9	0.2393 (8)	0.1892 (7)	0.8261 (2)	0.0605 (14)	
H9B	0.2074	0.2369	0.8645	0.073*	
C10	0.1074 (9)	0.2188 (8)	0.7765 (3)	0.0665 (14)	
H10A	−0.0132	0.2855	0.7822	0.080*	
C11	0.1489 (9)	0.1524 (7)	0.7186 (3)	0.0627 (13)	
C12	0.3281 (10)	0.0573 (7)	0.7125 (2)	0.0697 (15)	
H12D	0.3618	0.0133	0.6736	0.084*	
C13	0.4616 (9)	0.0235 (8)	0.7617 (2)	0.0657 (14)	
H13D	0.5812	−0.0441	0.7559	0.079*	
C14	0.0019 (11)	0.1849 (9)	0.6647 (3)	0.091 (2)	
H14A	0.0789	0.1720	0.6265	0.136*	
H14B	−0.0533	0.2980	0.6675	0.136*	
H14C	−0.1138	0.1055	0.6659	0.136*	

### Atomic displacement parameters (Å^2^)

**Table:**

	*U*^11^	*U*^22^	*U*^33^	*U*^12^	*U*^13^	*U*^23^
S1	0.0456 (10)	0.0627 (11)	0.0658 (10)	0.000	0.000	−0.0035 (10)
C2	0.051 (3)	0.062 (3)	0.048 (2)	0.001 (3)	0.001 (2)	−0.004 (2)
C3	0.046 (3)	0.085 (4)	0.062 (3)	0.005 (3)	0.002 (2)	−0.018 (3)
C4	0.056 (3)	0.063 (3)	0.055 (3)	0.005 (3)	0.002 (2)	−0.004 (3)
N5	0.059 (3)	0.057 (3)	0.057 (2)	0.002 (2)	−0.007 (2)	−0.006 (2)
C6	0.068 (3)	0.064 (4)	0.064 (3)	0.006 (3)	−0.017 (3)	−0.011 (3)
C7	0.151 (7)	0.063 (4)	0.082 (4)	−0.013 (4)	−0.040 (5)	0.012 (3)
C8	0.055 (3)	0.048 (3)	0.059 (3)	0.000 (2)	0.001 (2)	−0.005 (2)
C9	0.056 (3)	0.068 (3)	0.057 (3)	0.004 (3)	0.006 (2)	−0.009 (3)
C10	0.056 (3)	0.063 (3)	0.080 (3)	0.007 (3)	0.000 (3)	−0.005 (3)
C11	0.064 (3)	0.055 (3)	0.068 (3)	−0.009 (3)	−0.010 (3)	0.000 (3)
C12	0.080 (4)	0.069 (4)	0.059 (3)	0.000 (3)	−0.002 (3)	−0.009 (3)
C13	0.064 (3)	0.066 (3)	0.067 (3)	0.015 (3)	−0.003 (2)	−0.018 (3)
C14	0.098 (5)	0.087 (5)	0.088 (4)	−0.005 (4)	−0.032 (4)	0.008 (4)

### Geometric parameters (Å, º)

**Table:**

S1—C2	1.716 (5)	C8—C9	1.371 (7)
S1—C2^i^	1.716 (5)	C8—C13	1.387 (7)
C2—C3	1.350 (7)	C9—C10	1.370 (7)
C2—C4	1.446 (7)	C9—H9B	0.9300
C3—C3^i^	1.403 (10)	C10—C11	1.377 (8)
C3—H3B	0.9300	C10—H10A	0.9300
C4—N5	1.246 (6)	C11—C12	1.359 (8)
C4—H4A	0.9300	C11—C14	1.502 (8)
N5—C6	1.475 (7)	C12—C13	1.374 (8)
C6—C8	1.504 (7)	C12—H12D	0.9300
C6—C7	1.518 (9)	C13—H13D	0.9300
C6—H6B	0.9800	C14—H14A	0.9600
C7—H7B	0.9600	C14—H14B	0.9600
C7—H7C	0.9600	C14—H14C	0.9600
C7—H7D	0.9600		
			
C2—S1—C2^i^	91.5 (3)	C9—C8—C6	122.0 (5)
C3—C2—C4	127.6 (5)	C13—C8—C6	120.1 (5)
C3—C2—S1	111.2 (4)	C10—C9—C8	121.1 (5)
C4—C2—S1	121.1 (4)	C10—C9—H9B	119.5
C2—C3—C3^i^	113.0 (3)	C8—C9—H9B	119.5
C2—C3—H3B	123.5	C9—C10—C11	121.6 (5)
C3^i^—C3—H3B	123.5	C9—C10—H10A	119.2
N5—C4—C2	123.0 (5)	C11—C10—H10A	119.2
N5—C4—H4A	118.5	C12—C11—C10	117.0 (5)
C2—C4—H4A	118.5	C12—C11—C14	122.0 (6)
C4—N5—C6	116.4 (4)	C10—C11—C14	121.0 (6)
N5—C6—C8	110.3 (4)	C11—C12—C13	122.6 (5)
N5—C6—C7	107.9 (4)	C11—C12—H12D	118.7
C8—C6—C7	110.7 (5)	C13—C12—H12D	118.7
N5—C6—H6B	109.3	C12—C13—C8	119.9 (5)
C8—C6—H6B	109.3	C12—C13—H13D	120.0
C7—C6—H6B	109.3	C8—C13—H13D	120.0
C6—C7—H7B	109.5	C11—C14—H14A	109.5
C6—C7—H7C	109.5	C11—C14—H14B	109.5
H7B—C7—H7C	109.5	H14A—C14—H14B	109.5
C6—C7—H7D	109.5	C11—C14—H14C	109.5
H7B—C7—H7D	109.5	H14A—C14—H14C	109.5
H7C—C7—H7D	109.5	H14B—C14—H14C	109.5
C9—C8—C13	117.8 (5)		
			
C2^i^—S1—C2—C3	0.5 (3)	C7—C6—C8—C13	−100.2 (6)
C2^i^—S1—C2—C4	−177.0 (5)	C13—C8—C9—C10	0.9 (8)
C4—C2—C3—C3^i^	176.0 (5)	C6—C8—C9—C10	−175.9 (5)
S1—C2—C3—C3^i^	−1.3 (7)	C8—C9—C10—C11	−0.6 (9)
C3—C2—C4—N5	−171.3 (5)	C9—C10—C11—C12	−0.7 (9)
S1—C2—C4—N5	5.7 (7)	C9—C10—C11—C14	179.6 (6)
C2—C4—N5—C6	179.4 (4)	C10—C11—C12—C13	1.6 (9)
C4—N5—C6—C8	−132.6 (5)	C14—C11—C12—C13	−178.6 (6)
C4—N5—C6—C7	106.3 (6)	C11—C12—C13—C8	−1.4 (9)
N5—C6—C8—C9	−42.7 (7)	C9—C8—C13—C12	0.0 (8)
C7—C6—C8—C9	76.6 (7)	C6—C8—C13—C12	176.9 (5)
N5—C6—C8—C13	140.5 (5)		

Symmetry code: (i) *x*, −*y*+1, −*z*+2.
